# HPLC-ESI-qTOF-MS/MS Characterization, Antioxidant Activities and Inhibitory Ability of Digestive Enzymes with Molecular Docking Analysis of Various Parts of Raspberry (*Rubus ideaus* L.)

**DOI:** 10.3390/antiox8080274

**Published:** 2019-08-03

**Authors:** Lingfeng Wu, Yufeng Liu, Yin Qin, Lu Wang, Zhenqiang Wu

**Affiliations:** 1 College of Food Science and Engineering, Hainan University, Haikou 570228, China; 2 School of Biology and Biological Engineering, South China University of Technology, Guangzhou 510006, China; 3 School of Pharmaceutical Engineering, Guizhou Institute of Technology, Guiyang 550003, China; 4 Pan Asia (Jiangmen) Institute of Biological Engineering and Health, Jiangmen 529080, China

**Keywords:** *Rubus idaeus* L., phenolic compounds, HPLC-ESI-HR-qTOF-MS/MS, antioxidant activities, digestive enzymes inhibitors, molecular docking analysis

## Abstract

The anti-oxidative phenolic compounds in plant extracts possess multiple pharmacological functions. However, the phenolic characterization and in vitro bio-activities in various parts of raspberry (*Rubus idaeus* L.) have not been investigated systematically. In the present study, the phenolic profiles of leaves (LE), fruit pulp (FPE), and seed extracts (SE) in raspberry were analyzed by HR-HPLC-ESI-qTOF-MS/MS method, and their antioxidant activities and digestive enzymes inhibitory abilities were also investigated. The molecular docking analysis was used to delineate their inhibition mechanisms toward type II diabetes related digestive enzymes. Regardless of LE, FPE, or SE, 50% methanol was the best solvent for extracting high contents of phenolic compounds, followed by 50% ethanol and 100% methanol. The LE of raspberry displayed the highest total phenolic content (TPC) and total flavonoid content (TFC). A total of nineteen phenolic compounds were identified. The quantitative results showed that gallic acid, ellagic acid, and procyanidin C3 were the major constituents in the three extracts. The various parts extracts of raspberry all exhibited the strong antioxidant activities, especially for LE. Moreover, the powerful inhibitory effects of the three extracts against digestive enzymes (*α*-glucosidase and *α*-amylase) were observed. The major phenolic compounds of the three extracts also showed good inhibitory activities of digestive enzyme in a dose-dependent manner. The underlying inhibitory mechanisms of the main phenolic compounds against digestive enzymes were clarified by molecular docking analysis. The present study demonstrated that the various parts of raspberry had strong antioxidant activities and inhibitory effects on digestive enzymes, and can potentially prevent oxidative damage or diabetes-related problems.

## 1. Introduction

Nowadays, incidences of metabolic syndrome-related diseases have rapidly increased all over the world, such as oxidative damage, diabetes, obesity, hypertension, and cardiovascular disease [1]. Food nutritionists have revealed that oxidative stress and high-calorie diets was closely related to the development of chronic disease [2]. To some extent, chemical drugs have good curative effect for relieving these chronic diseases, but they may also bring seriously side effects and drug dependence to the human body. Polyphenolics, as one of the ubiquitous secondary metabolites in plants, have been proven to possess important physiological functions including anti-oxidant, anti-diabetes, anti-inflammatory, anti-carcinogenic, anti-obesity, and anti-proliferative activities [3,4]. Currently, many scientists have pointed out that phytochemicals (especially phenolics and flavonoids, etc.) from the plant-based foods may be used as natural antioxidants, which can inhibit oxidative damage. Moreover, long-term intake of plant-based foods enriched in polyphenolics compounds is conducive to prevent the occurrence of chronic diseases caused by oxidative stress [5,6]. 

Raspberry (*Rubus idaeus* L.) belongs to the *Rosaceae* family, which is widely cultivated in Asia, Europe, and North America [7]. Fruits of raspberry are widely consumed as fresh fruits, functional beverages, and fermented wine due to its attractive color, delicious taste, and excellent nutritional properties (enriched in anti-oxidative phenolics) [8]. The leaves of raspberry have also received considerable attention for its human health benefits including anti-oxidant, anti-diabetes, and anti-inflammatory activities, and have been made into tea product and its consumption has been also increasing [9,10,11]. Raspberry seeds are also good raw materials for food industries, but there is little research focused on the its phyto-constituents and bio-activities [12]. Qin et al. (2018) have reported that raspberry fruits and seeds include large amounts of anti-oxidative bound phenolics. During in vitro digestion, the released bound phenolics (non-extractable) possessed antioxidant activities and the inhibitory effect of *α*-glucosidase [13]. Moreover, different extraction methods for releasing the non-extractable phenolics in the leaves and seeds of raspberry have been investigated in our previous work [14]. To the best of our knowledge, the characterization and bio-activities (especially their antioxidant activities and the inhibitory properties on digestive enzymes) of phenolic extracts in different parts of raspberry have not been compared and investigated systematically.

The aim of the present work was to systematically investigate the phenolic profiles and in vitro antioxidant activities of leaves, fruit pulp, and seeds extracts of raspberry. Meanwhile, the inhibitory effects against digestive enzymes (*α*-glucosidase and *α*-amylase) were also evaluated. Importantly, the inhibition mechanisms of the main phenolic molecules toward digestive enzymes were investigated by molecular docking analysis. This work may supply important evidence for the comprehensive utilization of raspberry in food industries.

## 2. Materials and Methods

### 2.1. Chemicals and Reagents

The Folin–Ciocalteu phenol reagent and chemicals used for the antioxidant activity assay were purchased from Aladdin Industrial Corporation (Shanghai, China). Phenolic standards, trolox, *p*-Nitrophenyl-*α*-D-glucopyranoside (*p*-NPG, > 99.8%), porcine pancreatic *α*-amylase (13 U/mg), and *α*-glucosidase (≥ 58 U/mg solid) from *Saccharomyces cerevisiae* were all purchased from Sigma-Aldrich (St. Louis, MO, USA). Analytical-grade materials were purchased from Guangzhou Reagent Co. (Guangzhou, China). Formic acid and acetonitrile used for the HPLC analysis were of chromatography grade. Water was purified by the Milli-Q (Millipore, MA, USA) system and filtered through membranes with a pore size of 0.22 μm before use.

### 2.2. Materials

Raspberry was provided from Guishanhong Agricultural Development Co. Ltd., (Guiyang, Guizhou, China). The leaves, fruit pulp, and seeds of raspberry were separated, vacuum freeze-dried to remove the water, and then ground in a mechanic micromill (BJFSJ-150G, Shanghai, China) to fine powder, respectively. All samples were stored at −20 °C until use.

### 2.3. Extraction of Phenolic Compounds

One gram of the above sample powder was soaked with 10 mL of different extractions solvents (50% ethanol (EtOH), 100% EtOH, 50% methanol, 100% methanol, ethyl acetate and acetone), and then sonicated for 30 min at 40°C, 320 W. The mixture was subsequently filtered through a Whatman No. 1 paper.

### 2.4. Determination of Total Phenolic Content (TPC) and Flavonoid Content (TFC) 

TPC was measured according to the Folin–Ciocalteau method with gallic acid as the standard [15]. TPC were expressed as mg gallic acid equivalents (GAE)/g sample in dry weight (DW). TFC was determined based on the aluminium chloride colorimetric method with rutin as the standard [16]. TFC were expressed as mg rutin equivalents (RE)/g sample in DW. 

### 2.5. Phenolic Compositions Analysis by HPLC-ESI-HR-qTOF-MS/MS 

The phenolic compositions of the three parts extracts’ (extracted by 50% methanol) of raspberry were separated by using an HPLC system (Agilent 1200, CA, USA) equipped with a Diode Array Detector (DAD, Aglient, CA, USA). The analytical column was 250 mm × 4.6 mm, Zorbax Eclipse C_18_ plus column (5 μm, Aligent, CA, USA). Acetonitrile including 0.1% formic acid (phase A) and water including 0.1% formic acid (phase B) were used as the mobile phases. The gradient elution program was 0–5 min, 15% A; 5–20 min, 15–25% A; 20–30 min, 25–35% A; 30–40 min, 35–50% A; 40–50 min, 80% A; 50–55 min, 15% A, with a flow rate of 0.8 mL/min. The injection volume was 10 μL, temperature of the column was set to 30°C, and the UV/DAD were monitored from 200 to 600 nm. The HR-qTOF-MS/MS analysis was performed with a high-resolution time-of-flight (HR-qTOF) mass detector (maXis, Bruker, Billerica, MA, USA) in the negative or positive mode (4.0 kV). Mass spectra were recorded over the mass range *m/z* 100 to 1000. The acquired MS data were processed by Bruker Daltonics DataAnalysis software. The contents of the analytes were expressed as mg per g DW (Table S1).

### 2.6. Antioxidant Activities Assays

#### 2.6.1. DPPH Radical Scavenging Activity Assay

DPPH assay was measured based on the method described earlier [17]. The absorbance at 517 nm was recorded by a microplate reader (SpectraMax M5 Molecular Device, CA, USA). Trolox or Vc solution (5–100 μg/mL) were served as positive controls. The results were expressed as the percentage of inhibition in the following Equation (1).

(1) $$\mathrm{DPPH}\;\mathrm{radical}\;\mathrm{scavenging}\;\mathrm{activity}\;(\%)\;=\;\left(1-\frac{A_{s}-A_{b}}{A_{c}}\right)\times 100$$

where *A_s_* = the absorbance of the sample extracts with DPPH solution, *A_b_* = the absorbance of the sample extracts without DPPH solution, *A_c_* = the absorbance of DPPH solution. 

#### 2.6.2. ABTS Cation Radical Scavenging Activity Assay

ABTS assay was determined according to the method of Wang et al. (2017) [18]. The absorbance at 517 nm was recorded by the microplate reader. Trolox or Vc solution (5–100 μg/mL) was served as positive controls. The scavenging activity (%) was calculated by Equation (2).

(2) $${\mathrm{ABTS}}^{+}\;\mathrm{radical}\;\mathrm{scavenging}\;\mathrm{activity}\;(\%)\;=\;\left(1-\frac{A_{s}-A_{b}}{A_{c}}\right)\times 100$$

where *A_s_* = the absorbance of the extracts with ABTS^+^ solution, *A_b_* = the absorbance of the extracts without ABTS^+^ solutionl, *A_c_* = the absorbance of ABTS^+^ solution.

#### 2.6.3. Hydroxyl (OH^−^) Radical Scavenging Activity Assay

The scavenging activity of OH^−^ radicals was measured based on the method described by Liu et al., 2017 [19]. The reaction mixture included 100 μL of the extracts’ dilutions, 100 μL of 6 mM Fe_2_SO_4_ solution, and 100 μL of 2.4 mM H_2_O_2_. After 10 min of incubation at 25°C, the mixture was incubated with 100 μL of 6 mM salicylic acid at 25 °C for 30 min, then the absorbance at 510 nm was measured. A Trolox or Vc solution (5–100 μg/mL) served as the positive control. 

#### 2.6.4. Ferric Reducing/Antioxidant Power (FRAP) Assay

The FRAP assay was measured according to the method reported by Wong, Li, Cheng, and Chen (2006) [20]. A standard curve was constructed using the FeSO_4_·7H_2_O (0–1000 μM) as the reference standard. The FRAP values were expressed in mM ferrous sulfate equivalents Fe(Ⅱ)SE/g sample in DW (mM Fe(Ⅱ)SE/g DW).

### 2.7. Type II Diabetes Related Enzyme Inhibition Properties

#### 2.7.1. α-Glucosidase Inhibition Activity Assay

The inhibitory activity of *α*-glucosidase was performed as the previous reported method with modifications [21]. Briefly, 100 μL of the extracts’ dilutions and 100 μL of 1 U/mL *α*-glucosidase in 0.1 M phosphate buffer solution (pH 6.8) was mixed and pre-incubated at 37 °C for 10 min. Then, 100 μL of 5 mM *p*-NPG solution was added, and the reaction solution was incubated for another 20 min. The reaction was terminated by adding 500 μL of 0.2 M Na_2_CO_3_ solution. The absorbance at 405 nm was recorded. Acarbose was used as a positive control. The inhibitory potency (%) was calculated by Equation (3).

(3) $$\alpha -\mathrm{Glucosidase}\;\mathrm{inhibitory}\;\mathrm{potency}\;(\%)\;=\;\left[1-\frac{\Delta As}{\Delta Ac}\right]\times 100$$

where ΔA_s_ = A_extract+enzyme_ − A_extract_, ΔA_c_ = A_buffer+enzyme_ − A_buffer_.

#### 2.7.2. α-Amylase Inhibition Activity Assay

The inhibitory activity of *α*-amylase was carried out according to the literature with slight modification [22]. An amount of 200 μL of the extracts’ dilutions were mixed with 200 μL of *α*-amylase (0.5 mg/mL in PBS, pH = 6.8), and the mixture was pre-incubated at 37 °C for 15 min. Then, 400 μL of 2 mg/mL soluble starch solution was added to start the hydrolysis at 37 °C for 10 min. Afterwards, 1 mL of DNS reagent was added to stop the hydrolysis reaction, and the mixture was placed in boiling water bath for 5 min. When being cooled to room temperature, the absorbance at 540 nm was measured. The solution without *α*-amylase was used as the blank. The inhibitory potency (%) was calculated by Equation (4).

(4) $$\alpha -\mathrm{Amylase}\;\mathrm{inhibitory}\;\mathrm{potency}\;(\%)\;=\;\left[1-\frac{\Delta As}{\Delta Ac}\right]\times 100$$

where ΔA_s_ = A_extract+enzyme_ − A_extract_, ΔA_c_ = A_buffer+enzyme_ − A_buffer_.

### 2.8. Molecular Docking Analysis

The 2D conformers of the main phenolic standards (gallic acid, ellagic acid, and procyanidin C3) and acarbose (an anti-diabetic drug) were drawn by Chem 3D software, and the PDB formats of the *α*-glucosidase (PDB ID:3A4A) and *α*-amylase (PDB ID:1PPI) were downloaded from RCSB PDB (http://www.rcsb.org/pdb/home/home.do) [23]. Because the structural information of *α*-glucosidase from *S. cerevisiae* was not available, the homology structural (isomaltase, PDB ID: 3A4A) with high similarity of *α*-glucosidase was used as the template to perform the *α*-glucosidase docking analysis [23]. The molecular docking analysis of the main phenolic standards and acarbose to digestive enzymes was performed by the Surflex-Dock Geom (SFXC) mode using SYBYL-X 2.0 software package (Tripos, Inc., St. Louis, MO, USA). The docking procedure of small molecules to digestive enzymes is referred to in the literature [24]. Subsequently, a docking score file was generated and saved as the SD format. A C-Score (≥ 4) was selected as the credible results for the next docking analysis. The relevant docking parameters (e.g., T-Score, PMF-Score, D-Score, CHEM-Score, amino acid residues with active site, and hydrogen bond distances, etc.) may be used to reveal the inhibition mechanism of small molecules to digestive enzymes. The Surflex-Dock scoring function is a weighted sum of non-linear functions involving van der Waals surface distances between the appropriate pairs of exposed enzyme and ligand atoms.

### 2.9. Data Analysis

All the experiments were carried out in triplicate. Values were presented as the mean values ± standard deviation (SD). Analyses of variance and significance differences were analyzed by SPSS Statistics version 17.0 (IBM SPSS, Chicago, USA). 

## 3. Results and Discussion

### 3.1. Total Phenolic Content and Total Flavonoid Content

The choice of the extraction solvent is very important for the recovering of phenolic compounds in plant matrix [25]. Common solvents including ethanol, methanol, acetone, and ethyl acetate have been widely used for the extraction of nature antioxidants in plant matrix [26,27].

**Figure 1:**
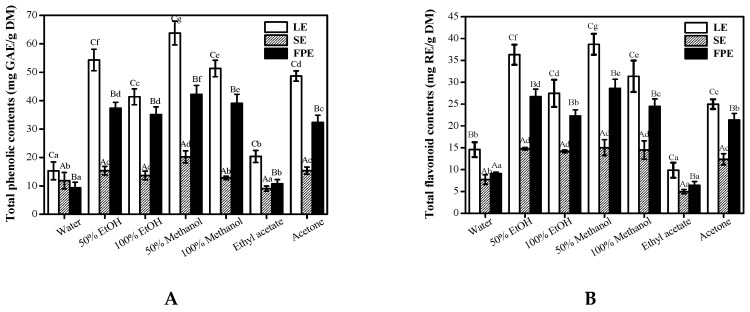
The effects of extraction solvents on total phenolic contents (**A**) and total flavonoid contents (**B**) in various parts of raspberry. LE, leaves extracts; SE, seeds extracts; FPE, fruit pulp extracts; EtOH, ethanol. Different lowercase letters (a–g) mean statistically significant differences following different extraction solvents. Different uppercase letters (A–C) mean statistically significant differences following different samples under same extraction solvents.

As seen from Figure 1, results of TPC and TFC showed that concentrations significantly varied between the different solvents in various parts of raspberry. Regardless of LE, FPE, or SE, 50% methanol/ethanol showed high efficiency in the extraction of total phenolic and flavonoid compounds, followed by 100% EtOH and 100% methanol. In contrast, water and ethyl acetate were the least efficient solvents for extracting the phenolics and flavonoids compounds. It is worth noting that 50% methanol was evidently better than 100% methanol for extracting phenolic compounds (*p* < 0.01), which may be due to that the extraction efficiency depends on the polarity of the compounds present in the samples [28,29]. P. López-Perea et al, (2019) also reported that 80% methanol showed higher efficiency in the extraction of phenolic compounds from wheat bran and barley husk than 100% methanol [30]. When extracted by 50% methanol solution, the highest TPC (63.79 ± 3.11 mg GAE/g DW) and TFC (38.68 ± 2.4 mg RE/g DW) were found in LE, followed by FPE (42.26 ± 3.11 mg GAE/g DW for TPC, 28.60 ± 2.12 mg RE/g DW for TFC), and comparatively low amounts in SE (20.25 ± 1.79 mg GAE/g DW for TPC, 15.03 ± 1.82 mg RE/g DW for TFC). Wanyo et al. (2014) reported that 64% ethanol was the most efficient phenolic compounds extraction solvent [31]. Djordjevic et al. (2011) showed that high concentrations of phenolic compounds in barley grain (*Hordeum vulgare*) were obtained with 70% ethanol [32]. Our results also confirmed the choice of solvent varieties and concentrations have very significant influences in the extraction efficiency of phenolic compounds. Therefore, in the next study, in order to investigate the phenolic compositions and their in vitro biological activities of various parts in raspberry, 50% methanol was chosen as the best extraction solvent.

### 3.2. HPLC-ESI-HR-qTOF-MS/MS Characterization and Quantification of Phenolic Compositions 

**Figure 2:**
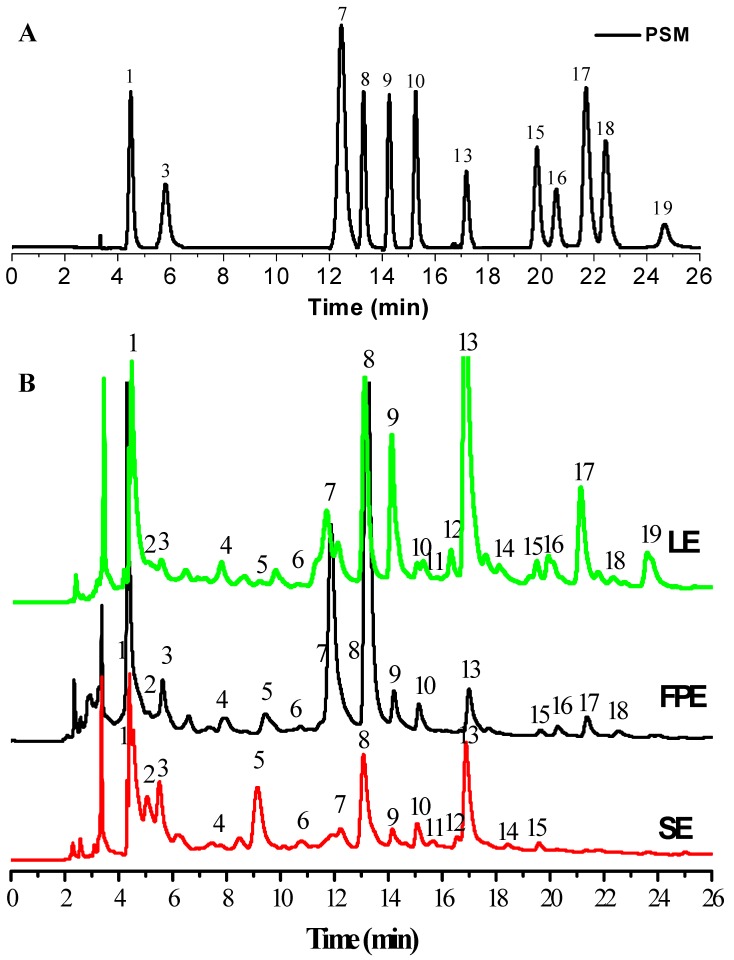
HPLC chromatograms (280 nm) of phenolic standards mixtures (**A**) and various parts extracts in raspberry (**B**). Peaks identification and their MS data are shown in Table 1. PSM, phenolic standard mixtures; 1, Gallic acid; 3, Epicatechin; 7, Procyanidin B2; 8, Procyanidin C3; 9, Ellagic acid pentoside; 10, Rutin; 13, Ellagic acid; 15, Quercetin 3-O-glucoside; 16, Avicularin; 17, Kaempferol-7-O-glucuronide; 18, Quercetin-7-O-glucuronide; 19, Kaempferol-3-O-glucuronide. LE, leaves extracts; SE, seeds extracts; FPE, fruit pulp extracts.

The phenolic compositions in different samples were identified by comparing their retention times and MS spectrum data with the authentic standards and reported data [14]. Table 1 and Figure 2 show the corresponding identification results of each peaks in HPLC chromatograms. Based on the MS/MS spectrum data, compound 1 displayed a [M-H]^−^ in *m/z* 169.1221, which was easily identified as gallic acid. Chlorogenic acid (compound 2) and epicatechin (compound 3) were identified by their UV spectrum and MS spectral data, respectively. Compounds 4, 5, and 7 showed the parent ions at 579 [C_30_H_26_O_12_+H]^+^ and its fragments ions at *m/z* 291.0503 [C_15_H_14_O_6_+H]^+^, which can be tentatively identified as three isomers of procyanidin dimers. Among them, compound 7 can be identified as procyanidin B2 according to MS spectrum data and the retention time of the standard. Compounds 6 and 8 showed the parent ions [M+H]^+^ at 867 and its MS/MS fragments ions at 579.1502 [C_30_H_26_O_12_+H]^+^ and 291.0155 [C_15_H_14_O_6_+H]^+^, respectively, which can be likely identified as two isomers of procyanidin trimer. By comparing the MS fragments information and the retention time of the standard, compound 8 can be identified as procyanidin C3. Ellagic acid pentoside was easily identified by the parent ion [M+H]^+^ at *m/z* 435 and its ion fragment at *m/z* 303.0142 [C_14_H_6_O_8_+H]^+^. Compound 10 was identified as rutin by the parent ion [M+H]^+^ at *m/z* 611 and its ions fragments *m/z* at 303.0563 [M-glc+H]^+^ and 163.1221 [M-C_15_H_10_O_7_]^+^. Compound 11 was kaempferol-galactoside-glucoside by its ions [M+H]^+^ at *m/z* 611 and two fragments ions at *m/z* 449.1338 [M−glc+H]^+^ and 287.0716 [C_15_H_10_O_6_+H]^+^. Compound 13 with [C_14_H_6_O_8_+H]^+^ ion at *m/z* 303 was easily identified as ellagic acid. Compounds 14 and 15 with [M+H]^+^ ions at *m/z* 465 giving MS/MS fragments ions *m/z* at 303 [C_15_H_10_O_7_+H]^+^ and 161 [M-C_15_H_10_O_7_+H]^+^ were very likely to be two isomers of quercetin glucosides. By comparing the retention time of two standard compounds, they were identified as quercetin-3-O-galactoside and quercetin-3-O-glucoside, respectively. Compound 16 with [M+H]^+^ ion *m/z* at 435 giving fragment ion at 303.0500 [C_15_H_10_O_7_+H]^+^ was identified as avicularin. Kaempferol-7-*O*-glucuronide (Compound 17) was easily identified by the [M+H]^+^ ion *m/z* at 463 and its MS/MS fragment ion at 287.0546 [C_15_H_10_O_6_+H]^+^. Quercetin-7-*O*-glucuronide (Compound 18) was identified by the ion [M+H]^+^ at *m/z* 479 and its fragment ion at 303.0506 [C_15_H_10_O_7_+H]^+^. Compound 19 was easily identified as kaempferol-3-*O*-glucuronide by the [M+H]^+^ ion at *m/z* 463 and its ion fragment at [C_15_H_10_O_7_+H]^+^.

The quantification results of fifteen phenolic compounds are shown in Table 2. The LE possessed the widest range of phenolic compositions, but also with the highest contents of individual phenolics. However, SE included the narrowest range of investigated compounds and the lowest contents of individual phenolics. Regardless of LE, FPE, or SE, gallic acid, ellagic acid, and procyanidin C3 were the most abundant of phenolic compounds. Meanwhile, the contents of gallic acid and ellagic acid in LE reached up to 539.42 ± 2.09 μg/g DW and 527.26 ± 3.27 μg/g DW. Their contents in FPE were 339.45 ± 2.17 μg/g DW and 95.42 ± 0.53 μg/g DW, respectively, which were significantly lower than those in LE. The contents of procyanidin B2 (10.72 ± 0.07 μg/g DW) and procyanidin C3 (252.37 ± 0.05 μg/g DW) in FPE were evidently higher than those in LE or SE. Particularly, some flavonols compounds (quercetin-3-glucoside, kaempferol-7-*O*-glucuronide, quercetin-7-*O*-glucuronide and kaempferol-3-*O*-glucuronide) except for rutin and avicularin were only found in LE and SPE of raspberry. Meanwhile, it can be found that there also existed high contents of some invididual phenolic compounds in SE, such as gallic acid (127.15 ± 3.21 μg/g DW), procyanidin C3 (29.12 ± 0.11 μg/g DW) and ellagic acid (48.32 ± 0.23 μg/g DW), which showed that SE may be used as a good food ingredient enriched in phenolic compounds. Qin et al. (2018) have confirmed that high levels of gallic acid and ellagic acid existing in the form of bound phenolics were found in raspberry fruit and seed extracts, which was consistent with the results of our study [13].

### 3.3. Antioxidant Activities

In order to fully reflect the antioxidant capacity of the samples extracts, four well-known chemical test methods including DPPH, ABTS^+^, and OH^−^ free radical scavenging activities and ferric reducing antioxidant activity (FRAP) were used to perform the antioxidant activity assays. 

**Figure 3:**
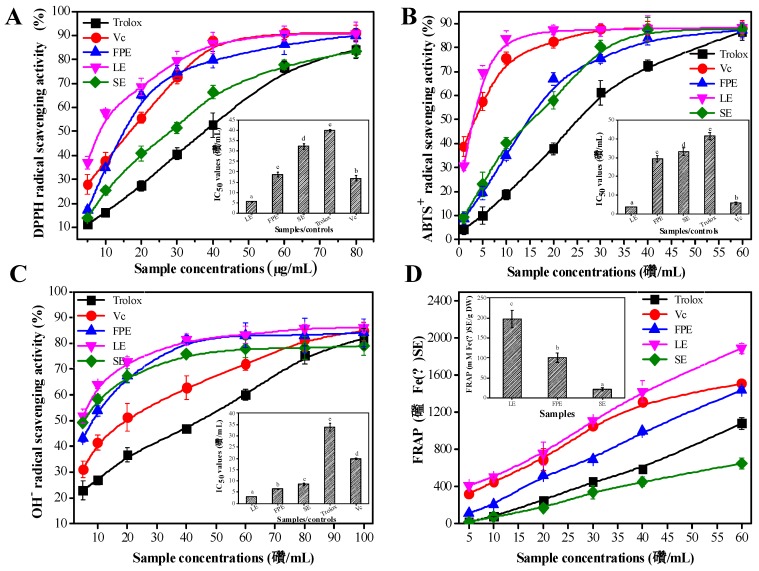
The antioxidant activities of various parts’ extracts of raspberry and the positive controls (Vc and Trolox): DPPH (**A**), ABTS^+^ (**B**), and OH^−^ radical scavenging activity (**C**) and Ferric reducing antioxidant power (FRAP) (**D**). The insets of Figure 3A–D represent the corresponding IC_50_ values of the samples/controls. LE, leaves extracts; FPE, fruit pulp extracts; SE, seed extracts. Vc, ascorbic acid. Different lowercase letters (a–e) mean statistically significant differences following different samples.

It was found that LE exhibited the strongest antioxidant activity, followed by FPE, and SE performed the weakest antioxidant activity. All of the samples’ extracts showed the antioxidant activities in a concentration-dependent manner (Figure 3A–D). The insets of Figure 3A–C show the corresponding IC_50_ values of the samples/controls. Notably, the lower the IC_50_ values indicated, the stronger the antioxidant activity. For the DPPH assay, the IC_50_ value of LE (5.60 ± 0.31 μg/mL) was lower than that of Vc (16.80 ± 1.31 μg/mL) and Trolox (39.90 ± 0.67 μg/mL), about one-third of that of FPE (18.71 ± 1.35 μg/mL), and one-sixth of that of SE (32.33 ± 1.42 μg/mL). LE also exhibited the strongest ABTS^+^ free radical scavenging activity. The IC_50_ value of LE (3.70 ± 0.17 μg/mL) was lower than that of Trolox (41.50 ± 1.97 μg/mL), FPE (29.43 ± 1.83 μg/mL), and SE (33.15 ± 2.19 μg/mL). Meanwhile, three various parts’ extracts in raspberry (LE, FPE and SE) also exhibited the strong OH^−^ free radical scavenging activity, and their corresponding IC_50_ values were 3.01 ± 0.13 μg/mL, 6.40 ± 0.27 μg/mL, and 8.61 ± 0.52 μg/mL, respectively. The IC_50_ value of LE was also lower than that of Vc (19.83 ± 0.37 μg/mL) and Trolox (33.91 ± 1.82 μg/mL). The insets of Figure 3A–C showed the results of FRAP assay. As is well known, higher FRAP values represent stronger anti-oxidant activity. The FRAP value of LE (198.32 ± 21.72 mM/g DW) was also higher than that of FPE (100.81 ± 12.31 mM/g DW) and SE (21.92 ± 3.72 mM/g DW).

The correlation co-efficient analysis results between the phenolic contents at different concentrations and antioxidant activities of the three extracts clearly verified that there was a good correlation between these two parameters (DPPH vs. TPC, *r* = −0.807, *p* < 0.01; ABTS vs. TPC, *r* = −0.875, *p* < 0.01; OH^−^ vs. TPC, *r* = −0.792, *p* < 0.01), suggesting that the phenolic compounds significantly contributed to the antioxidant activities of three samples extracts. Qin et al. (2018) reported that the antioxidant activities of raspberry fruits and seeds extracts were strongly correlated with the released phenolic contents during in vitro digestion [13]. Wang et al. (2019) also confirmed that the released bound phenolics of raspberry leaves and seeds treated with different methods were responsible for their antioxidant activities [14]. In the present study, gallic acid, ellagic acid, and procyanidin C3 were the major phenolic compounds in different parts of raspberry. Many researches have verified that these three phenolics possess strong antioxidant activity [33]. Malinda et al. (2017) reported that gallic acid and ellagic acid showed strong DPPH radical scavenging activity with IC_50_ values of 2.24 μg/mL and 4.80 μg/mL, respectively [34]. Bialonska et al. (2009) have also verified that ellagic acid displayed no significant differences in the antioxidant activity with Vc by in vivo testing [35]. Because LE possessed higher contents of these main phenolic compounds (especially for gallic acid and ellagic acid) than FPE and SE, thus, LE showed the strongest antioxidant capacity, followed by FPE. The results of the above studies further revealed that the phenolic compounds of various parts in raspberry may contribute evidently to their antioxidant activities.

### 3.4. Type II Diabetes Related Enzymes Inhibitory Activities

Alpha-glucosidase or *α*-amylase, two key digestive enzymes in the digestive tract, can break down macromolecular carbohydrates into monosaccharide/glucose. As well known, excess glucose accumulates in the blood instead of being used for energy, which may cause type II diabetes. Many researches have confirmed that phenolic compounds may bind the amino acid residues with the active sites of digestive enzymes into complex formation by hydrogen bonding, and thereby inhibit the catalytic reaction of digestive enzymes on carbohydrates [36]. Therefore, the phenolic fractions in various parts of raspberry on the inhibition of digestive enzymes were used to evaluate their potential hypoglycemic effect.

**Figure 4:**
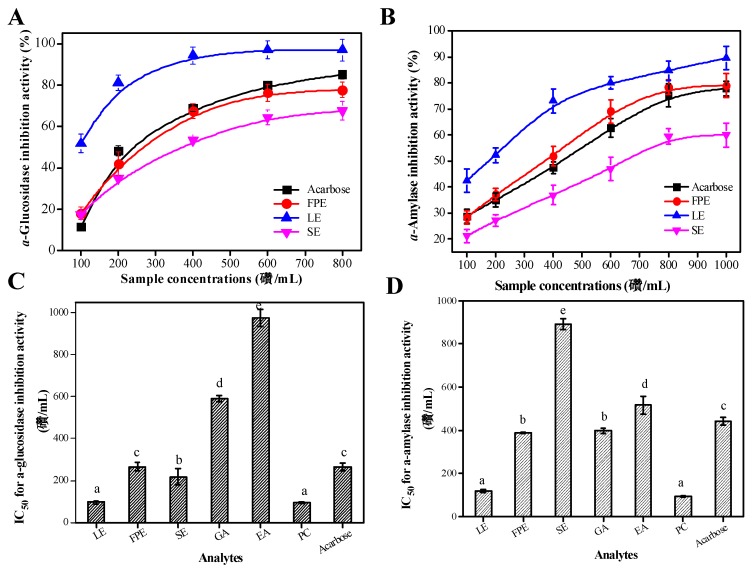
The digestive enzymes inhibitory abilities of various parts extracts in raspberry and acarbose: α-glucosidase inhibitory activity (**A**) and α-amylase inhibitory activity (**B**). Figure 4C,D represents the corresponding IC_50_ values of the samples/controls. LE, leaves extracts; FPE, fruit pulp extracts; SE, seed extracts; GA, gallic acid; EA, ellagic acid; PC, procyanidin C3. Different lowercase letters (a–e) mean statistically significant differences following different samples.

Figure 4 exhibits that the various parts of raspberry extracts tended to be strong inhibitors of type II diabetes-related enzymes. It can be found that the samples’ extracts or acarbose all showed the inhibition activity of digestive enzymes in a concentration-dependent manner (Figure 4A,B). Figure 4C,D show the IC_50_ values of the samples extracts or the main individual phenolic compounds on the inhibition effects on digestive enzymes. Similarly, the lower IC_50_ values indicated the stronger inhibition activity of digestive enzymes. The *α*-glucosidase inhibition activity of LE (IC_50_ = 96.50 ± 7.71 μg/mL) was evidently stronger than that of FPE (IC_50_ = 265.41 ± 20.7 μg/mL) and SE (IC_50_ = 218.5 ± 17.53 μg/mL) (*p* < 0.01). However, the *α*-glucosidase inhibition potency of acarbose (IC_50_ = 267.47 ± 19.72 μg/mL) was lower than that of LE (*p* < 0.05). FPE also possessed good inhibition activity against *α*-glucosidase, but there is no statistically significant difference with acarbose (*p* > 0.05). Figure 4C presents that the IC_50_ value for *α*-glucosidase inhibition activity of PC was 93.37 ± 5.79 μg/mL, which was significantly lower than that of GA (IC_50_ = 590.34 ± 15.71 μg/mL) and EA (IC_50_ = 976.32 ± 41.72 μg/mL) (*p* < 0.01). For *α*-amylase inhibition activity assay, LE have the lowest IC_50_ values (IC_50_ = 118.42 ± 2.79 μg/mL), followed by FPE (IC_50_ = 388.27 ± 2.47 μg/mL) and SE (IC_50_ = 891.12 ± 25.71 μg/mL). Moreover, the IC_50_ value of acarbose (IC_50_ = 442.23 ± 19.74 μg/mL) was higher than that of LE (*p* < 0.05), which indicates that LE may be used as a potential good anti-diabetic resource. Among the three main phenolic compounds, PC showed the strongest *α*-amylase inhibition activity (IC_50_ = 92.31 ± 3.51 μg/mL), followed by EA (IC_50_ = 516.73 ± 25.29 μg/mL), and GA (IC_50_ = 397.37 ± 12.37 μg/mL) (*p* < 0.01). 

The correlation analysis results between the TPC and two digestive enzymes inhibition potency clearly verified that there was a good positive correlation between these two parameters (*α*-glucosidase inhibition potency vs. TPC, *r* = 0.781, *p* < 0.05; *α*-amylase inhibition potency vs. TPC, *r* = 0.854, *p* < 0.01). Many researches have confirmed that phenolic-rich extracts from leaf-tea, edible fruits, and natural products possess good inhibitory ability on digestive enzymes [23,37]. Wang et al. (2018) have confirmed that procyanidin C3 possessed strong *α*-glucosidase inhibitory capacity [38]. Zhang et al. (2010) reported that gallic acid, anthocyanins, and rutin in raspberry have strong *α*-glucosidase inhibitory capacity, but ellagic acid possessed the weakest inhibitory ability of *α*-glucosidase, which was consistent with the results of our study [39].

### 3.5. Molecular Docking Results

**Figure 5:**
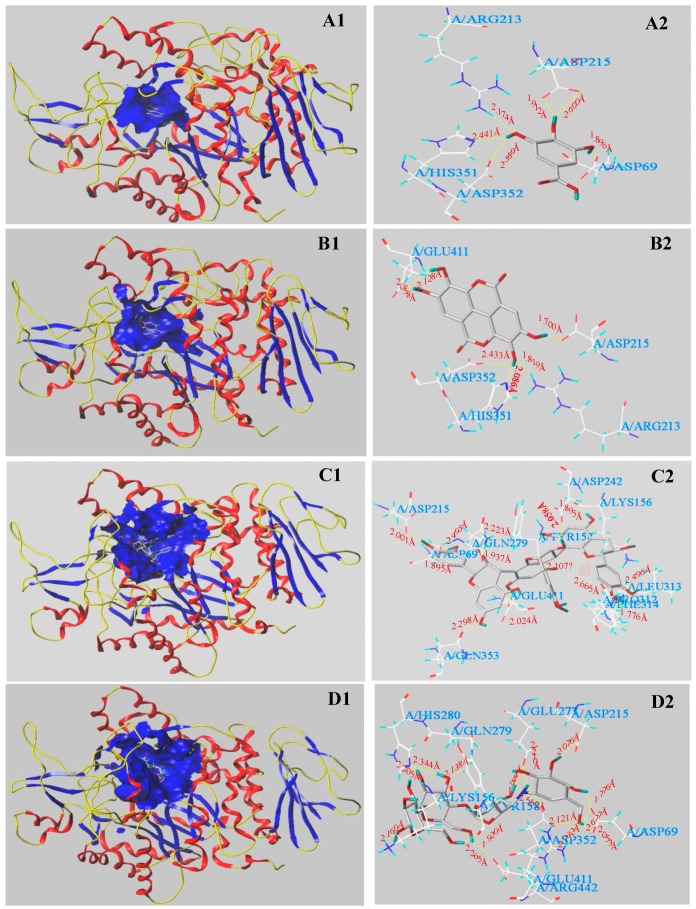
Molecular docking of the main three phenolic compounds and acarbose with the *α*-glucosidase. The 3D docking structures of three main phenolic compounds and acarbose were inserted into the hydrophobic cavity of the *α*-glucosidase (blue): gallic acid (**A1**); ellagic acid (**B1**); procyanidin C3 (**C1**); acarbose (**D1**). The conformation of active molecules interactions with amino acid residues in the active site of *α*-glucosidase: gallic acid (A2), ellagic acid (B2), procyanidin C3 (C2), and acarbose (D2) with residues in the active sites of the *α*-glucosidase, respectively. The dashed line stands for hydrogen bonds.

Molecular docking analysis was further done to analyze the digestive enzymes inhibitory mechanisms of the main phenolic compounds including GA, EA, and PC. The results clearly revealed that the structures of phenolic compounds significantly affect their inhibitory effects on *α*-glucosidase or *α*-amylase. Table 3 and Figure 5 show the molecular docking results with regard to interactions between *α*-glucosidase and several main phenolic molecules/acarbose binding. From Table 3, the molecular docking C-Scores values of those several molecules/acarbose were all ≥ 4, which indicates credible docking results. Figure 5 shows that GA interacted with the active sites of *α*-glucosidase and formed six H-bonds (yellow dotted line) with five amino acid residues (Asp 69, Arg 213, Asp 215, Asp 352, and His 351). The distances of H-bonds ranged from 1.896 Å to 2.600 Å. EA formed six H-bonds (the shortest distance was 1.700 Å and the longest distance was 2.433 Å) with five amino acid residues (Asp 215, Asp 352, Arg 213, Glu 411, and His 351). PC formed thirteen H-bonds within 4 Å (the distances ranged from 1.776 Å to 2.665 Å) with eleven amino acid residues (Asp 69, Asp 215, Asp 242, Glu 411, Gln 279, Gln 353, Leu 313, Lys 156, Tyr 158, The 314 and Pro 312). However, it was found that acarbose formed sixteen H-bonds within 4 Å (distances ranged from 1.776 Å to 2.732 Å) with ten amino acid residues (Asp 69, Asp 215, Asp 352, Arg 442, Glu 273, Glu 411, Gln 279, Tyr 158, Lys 156, and His 280). Some amino acid residues (Asp 69, Asp 215, and Asp 352) of α-glucosidase at least interacted with the above three investigated molecules, suggesting that these amino acid residues may play important roles in exerting the catalytic reaction of *α*-glucosidase.

**Figure 6:**
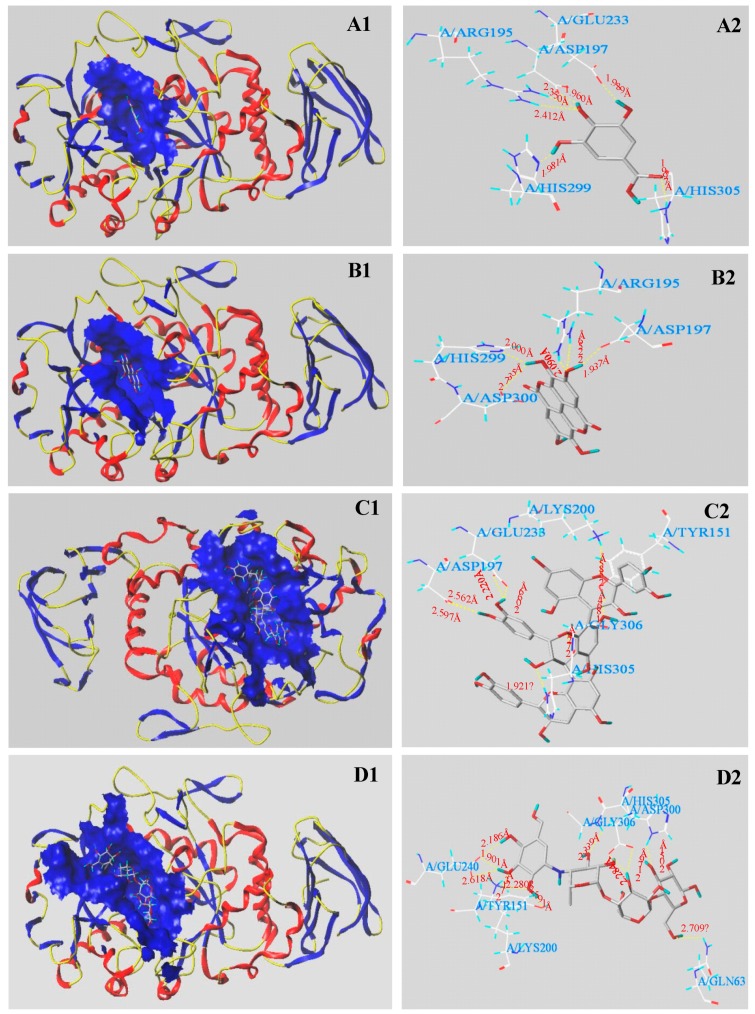
Molecular docking of the main three phenolic compounds and acarbose with *α*-amylase. The 3D docking structures of three phenolic compounds and acarbose were inserted into the hydrophobic cavity of *α*-amylase (blue): gallic acid (**A1**); ellagic acid (**B1**); procyanidin C3 (**C1**); acarbose (**D1**). The conformations of active molecules interactions with amino acid residues in the active site of *α*-amylase: gallic acid (**A2**), ellagic acid (**B2**), procyanidin C3 (**C2**), and acarbose (**D2**) with residues in the active sites of the *α*-amylase, respectively. The dashed line stands for hydrogen bonds.

Table 3 and Figure 6 show the molecular docking results of *α*-amylase with the investigated phenolic molecules. The C-Scores of three main phenolic molecules and acarbose were all ≥ 4. Figure 6 shows that six H-bonds (yellow dotted line) were formed between the active site of *α*-amylase and gallic acid. The five amino acid residues with the active site were Asp 197, Arg 195, Glu 233, His 299, and His 305, respectively. The average distance of six H-bonds was 2.115 Å. Ellagic acid formed H-bonds with four amino acid residues with the active site, namely Asp 197, Asp 300, Arg 195, and His 299. The distances of H-bonds ranged from 1.937 Å to 2.735 Å. However, procyanidin C3 formed nine H-bonds with the active sites of six amino acid residues (Asp 197, Glu 233, Gly 306, Lys 200, Tyr 155, and His 305). The shortest distance was 1.864 Å and the longest distance was 2.724 Å. It was found that acarbose formed eleven H-bonds (the distances ranged from 1.776 Å to 2.732 Å) with seven amino acid residues, namely, Asp 300, Gln 63, Glu 240, Gly 306, Tyr 151, Lys 200, and His 305.

It can be found that the numbers and distances of the H-bonds play important roles in exerting the catalytic reaction of the complex of digestive enzymes and ligands, and thereby cause the differences in inhibitory activity of digestive enzymes. Regardless of acarbose docked with *α*-glucosidase or *α*-amylase, the higher numbers of H-bonds and amino acid residues with active site were formed in the complex of digestive enzymes and acarbose. As a result, acarbose showed very good inhibitory effect of *α*-glucosidase and *α*-amylase. The numbers of H-bonds and amino acid binding sites formed by the two phenolic molecules (GA and EA) docked with *α*-glucosidase were equal, but there existed significant differences in inhibitory capacities of *α*-glucosidase. It may be due to that different interaction sites (amino acid residues) existed in between these molecules and *α*-glucosidase. Both of GA and EA all interacted with the amino acid residues His 351, Asp 215, Asp 352, and Arg 213 of *α*-glucosidase. EA also interacted with the amino acid residue Glu 411 of *α*-glucosidase. Some researchers have confirmed that some active sites (Glu 411) of *α*-glucosidase may inhibit the catalytic activity of this enzyme [37]. Consequently, ellagic acid showed the weakest *α*-glucosidase inhibitory effect. At least three investigated molecules formed H-bonds with the amino acid residues of Asp 69, Asp 215, and Asp 352, which may exert its *α*-glucosidase inhibitory effect. Hua et al. (2018) have also reported that Asp 69, Asp 215, and Arg 442 were the important residues involved in H-bond formation during the binding with *α*-glucosidase [37]. Zhang et al. (2018) also reported that the binding active sites (Asp 215 and Asp 352) in between the ligands and *α*-glucosidase played important roles in exert its *α*-glucosidase inhibitory effect [23]. Similarly, the order of amino acid residues numbers formed by four molecules (GA, EA, PC, and Acarbose) docked with *α*-amylase was: Acarbose (7) = PC (7) > GA (5) > EA (4). The order of H-bonds number was: Acarbose (11) > PC (9) > GA (6) > EA (5). Consequently, the order of the docking T-Score was: Acarbose > PC > GA > EA, which was consistent with the results of *α*-amylase inhibition activity. Moreover, at least three investigated molecules interacted with the amino acid residues Asp 197 and His 305 of *α*-amylase, indicating that these two amino acid residues may play critical roles in the catalytic reaction of *α*-amylase. Many reports have confirmed that the amino acid residues Asp 197 and His 305 played critical roles in the catalytic reaction of *α*-amylase [40,41]. The mechanisms of the digestive enzymes inhibitory activities of these compounds possibly involve the binding of compounds with the catalytic sites of digestive enzymes [37]. The results demonstrated that the hydrogen bonds and the binding residues with active sites have important effects on these digestive enzymes activities.

## 4. Conclusions

The solvents have significant impacts on the extraction of total phenolics and total flavonoids in various parts of raspberry. Fifty percent methanol was the best solvent for extracting high contents of phenolic and flavonoid compounds. The LE in raspberry displayed the highest TPC and TFC. A total of 19 phenolics compounds were identified. Gallic acid, ellagic acid, and procyanidin C3 were the main phenolic compositions existing in various parts of raspberry. Higher levels of phenolic compounds in raspberry showed the stronger anti-oxidant activities and inhibitory potency of digestive enzymes. The major phenolic compounds that were found in various parts of raspberry all showed good digestive enzyme inhibitory activities, especially for PC. Molecular docking analysis revealed the underlying inhibition mechanisms of these three main phenolic compounds against digestive enzymes, and the theoretical analysis data explained the experimental results very well.

## Figures and Tables

**Table 1:** Identification of phenolic compositions in various parts of raspberry by HPLC-ESI-HR-qTOF-MS/MS method.

Peak No.	Retention Time (min)	λ_max_ (nm)	Molecular ion (*m/z*)	MS (*m/z*)	Mw	Formula	Error (ppm)	Compounds (Abbreviation)	Reference	LE	FPE	SE
1	4.31	215, 271	169.2101[M-H]^−^	169.1221	170	C_7_H_6_O_5_	−0.7	Gallic acid (GA)	Standard	√	√	√
2	5.12	210, 268	353.2410[M+H]^−^	191.0121, 98.9212	354	C_16_H_17_O_9_	1.2	Chlorogenic acid	MS/MS			√
3	5.86	208, 279	291.0869 [M+H]^+^	291.0869, 209.1545, 138.1423	290	C_15_H_14_O_6_	−0.4	Epicatechin	Standard	√	√	√
4	7.87	215, 280	579.1503[M+H]^+^	579.1503, 291.0708	578	C_30_H_26_O_12_	2.7	Procyanidin dimer 1	MS/MS	√	√	
5	9.17	214, 280	579.1496[M+H]^+^	579.1496, 291.0503	578	C_30_H_26_O_12_	0.5	Procyanidin dimer 2	MS/MS		√	√
6	10.78	218, 284	867.2129 [M+H]^+^	867.2131, 563.1548, 291.0294	866	C_45_H_38_O_18_	2.2	Procyanidin trimer 1	MS/MS			√
7	11.98	220, 285	579.1514 [M+H]^+^	579.1514, 447.0508, 303.0134, 291.032	578	C_30_H_26_O_12_	0.3	Procyanidin B2	Standard	√	√	
8	13.19	225, 280	867.2140 [M+H]^+^	867.2131, 579.1502, 291.0155,185.0085	866	C_45_H_38_O_18_	0.2	Procyanidin C3 (PC)	Standard	√	√	√
9	14.27	253, 358	435.0564 [M+H]^+^	435.0564, 303.0142, 185.0085	434	C_19_H_14_O_12_	0.9	Ellagic acid pentoside	Standard	√	√	√
10	15.13	253, 354	611.1607 [M+H]^+^	611.1607, 449.0557, 303.0563	610	C_27_H_30_O_16_	−2.5	Rutin	Standard	√	√	√
11	15.87	254, 351	611.1607 [M+H]^+^	611.1254, 449.1338, 435.0557, 287.0716	610	C_27_H_30_O_16_	−2.5	kaempferol-galactoside-glucoside	MS/MS	√		
12	16.81	257, 353	867.2147 [M+H]^+^	611.1607, 479.0557, 303.0515, 209.1547	610	C_27_H_30_O_16_	1.7	Quercetin-3-O-glucuronide-arabinoside	MS/MS	√		√
13	16.95	256, 351	303.0501 [M+H]^+^	303.0501, 193.1221	302	C_14_H_6_O_8_	−0.3	Ellagic acid (EA)	Standard	√	√	√
14	18.21	254, 359	465.1032 [M+H]^+^	465.1032, 303.0498	464	C_21_H_20_O_12_	−0.5	Quercetin 3-O-galactoside	MS/MS	√		√
15	19.67	254, 356	465.1037 [M+H]^+^	465.1037, 303.0506	464	C_21_H_20_O_12_	−0.7	Quercetin 3-O-glucoside	Standard	√	√	√
16	21.57	262,391	435.0924 [M+H]^+^	435.0924, 303.0500, 219.1754	434	C_20_H_18_O_11_	−0.2	Avicularin	Standard	√	√	
17	20.42	253, 357	463.0807 [M+H]^+^	463.0807, 287.0546, 133.1526	462	C_21_H_18_O_12_	−0.6	Kaempferol-7-O-glucuronide	Standard	√	√	
18	22.72	254, 351	479.0827 [M+H]^+^	479.0829, 303.0506	478	C_21_H_18_O_13_	0.2	Quercetin-7-O-glucuronide	Standard	√	√	
19	23.51	257, 363	463.0878 [M+H]^+^	463.0878, 317.0123, 287.0557	462	C_21_H_18_O_12_	−1.4	Kaempferol-3-O-glucuronide	Standard	√		

Notes: LE, leaves extracts; FPE, fruit pulp extracts; SE, seed extracts; GA, gallic acid; EA, ellagic acid; PC, procyanidin C3.

**Table 2:** Contents of the main individual phenolics of various parts in raspberry.

Analytes	Contents (μg/g DW)		
	LE	FPE	SE
Gallic acid	539.42 ± 2.09c	339.45 ± 2.17b	127.15 ± 3.21a
Epicatechin	3.47 ± 0.02b	0.41 ± 0.07a	0.32 ± 0.12a
* Procyanidin dimer 1	6.71 ± 0.07c	4.39 ± 0.05b	2.13 ± 0.09a
^*^ Procyanidin dimer 2	1.79 ± 0.05a	2.07 ± 0.03b	13.35 ± 1.12c
Procyanidin B2	6.82 ± 0.12b	21.72 ± 0.07c	0.17 ± 0.02a
^#^ Procyanidin trimer 1	N.D.	2.34 ± 0.01a	3.25 ± 0.03a
Procyanidin C3	149.17 ± 0.01b	252.37 ± 0.05c	29.12 ± 0.11a
Ellagic acid pentoside	67.88 ± 0.12c	12.82 ± 0.09b	5.87 ± 0.11a
Rutin	2.53 ± 0.16a	5.07 ± 0.07b	4.89 ± 0.15b
Ellagic acid	527.26 ± 3.27c	95.42 ± 0.53b	48.32 ± 0.23a
Quercetin 3-glucoside	7.39 ± 0.03c	4.35 ± 0.02b	1.57 ± 0.23a
Avicularin	35.87 ± 0.12b	16.73 ± 0.09a	N.D.
Kaempferol-7-O-glucuronide	21.31 ± 0.01b	14.31 ± 0.02a	N.D.
Quercetin-7-O-glucuronide	2.32 ± 0.05a	2.14 ± 0.07a	N.D.
Kaempferol-3-O-glucuronide	5.47 ± 0.02	N.D.	N.D.

Different lowercase letters (a–c) mean statistically significant differences following different samples (*p* < 0.05). N.D., not detected; LE, leaves extracts; FPE, fruit pulp extracts; SE, seed extracts. ^*^ Procyanidin dimer and ^#^ procyanidin trimer were quantified by procyanidin B2 and procyanidin C3, respectively.

**Table 3:** The analysis results of the main phenolic molecules and acarbose dockings into *α*-glucosidase or *α*-amylase ligands.

Digestive Enzymes	Main Phenolics	C-Score	T-Score	PMF-Score	CHEM-Score	G-Score	D-Score
***α*-Glucosidase**	GA	4	3.86	−117.262	−15.181	−201.051	−108.348
	EA	5	1.66	−143.165	−25.320	−169.858	−134.180
	PC	4	5.58	−169.841	−20.602	−259.179	−186.583
	Acarbose	4	11.50	−267.829	−18.801	−399.410	−268.642
***α*-Amylase**	GA	5	4.61	−100.463	−14.772	−137.871	−77.144
	EA	5	3.56	−108.516	−19.079	−156.583	−100.861
	PC	5	6.74	−157.306	−26.662	−326.221	−178.056
	Acarbose	5	7.07	−174.749	−7.459	−311.510	−211.197

Notes: GA, gallic acid; EA, ellagic acid; PC, procyanidin C3.

## Supplementary Materials

The following are available online at https://www.mdpi.com/2076-3921/8/8/274/s1, Table S1: Regression equation, R2, LOD, and LOQ results of the main individual phenolics of various parts in raspberry.

## Abbreviations

LE	leaves extracts
SE	seed extracts
FPE	fruit pulp extracts
DPPH	1,1-Diphenyl-2-picrylhydrazyl radical
ABTS	2-azino-bis (3-ethylbenzothiazoline-6-sulfonic acid) diammonium salt
TPTZ	2,4,6-tris(2-pyridyl)-s-triazine
FRAP	ferric reducing anti-oxidant power
*p*-NPG	*p*-Nitrophenyl-*α*-D-glucopyranoside
TPC	total phenolic content
TFC	flavonoid content
GA	gallic acid
EA	ellagic acid
PC	procyanidin C3
Vc	ascorbic acid
DW	dried weight
HPLC-ESI-HR-qTOF-MS/MS	High performance liquid chromatography-electrospray ionization-high resolution-quadrupole time of flight-tandem mass spectrometry
PBS	phosphate buffer solution
GAE	gallic acid equivalents
RE	rutin equivalents
