# Supplementary material for: HPLC-ESI-qTOF-MS/MS Characterization, Antioxidant Activities and Inhibitory Ability of Digestive Enzymes with Molecular Docking Analysis of Various Parts of Raspberry (Rubus ideaus L.)
Source: Antioxidants (Basel). 2019 Aug 3;8(8):274. doi: 10.3390/antiox8080274 (PMC6719955; doi:10.3390/antiox8080274)
Supplement: Supplementary file 1 [file antioxidants-08-00274-s001.pdf]

Supplementary Material Table S1 Regression equation, R^2, LOD, and LOQ results of the main individual phenolics of various parts in raspberry

Analytes	[a]Calibration curve	R^2	[b]LOD (mg/L)	[b]LOQ (mg/L)
Gallic acid	$Y=7.8332\times10^{-6}X+0.0067$	0.9994	0.46	0.79
Epicatechin	$Y=5.9871\times10^{-6}X+0.0091$	0.9978	0.32	0.56
Procyanidins B2	$Y=4.2983\times10^{-6}X+0.0056$	0.9989	0.45	0.67
Procyanidins C3	$Y=3.7982\times10^{-6}X+0.0052$	0.9971	0.67	0.71
Ellagic acid pentoside	$Y=7.6245\times10^{-6}X+0.0031$	0.9991	0.57	0.62
Rutin	$Y=2.7981\times10^{-6}X+0.0019$	0.9993	0.23	0.35
Ellagic acid	$Y=3.0157\times10^{-6}X+0.0073$	0.9992	0.37	0.42
Quercetin 3-glucoside	$Y=7.2459\times10^{-6}X+0.0051$	0.9995	0.19	0.31
Avicularin	$Y=7.1982\times10^{-6}X+0.0049$	0.9934	0.54	0.63
Kaempferol-7-O-glucuronide	$Y=6.9137\times10^{-6}X+0.0097$	0.9979	0.78	0.87
Quercetin-3-O-glucuronide	$Y=6.9819\times10^{-6}X+0.0057$	0.9992	0.63	0.72
Kaempferol-3-O-glucuronide	$Y=6.4952\times10^{-6}X+0.0071$	0.9992	0.34	0.45

[a] X representing the peak area; Y representing the standard concentration

[b] LOD, limit of detection; LOQ, limit of quatification
